# Antioxidant Activity of Bilirubin in Micellar and Liposomal Systems Is pH-Dependent

**DOI:** 10.3390/antiox13040426

**Published:** 2024-03-30

**Authors:** Paweł Przybylski, Michał Żebrowski, Wojciech Witkowski, Martyna Cybularczyk-Cecotka, Grzegorz Litwinienko

**Affiliations:** Faulty of Chemistry, University of Warsaw, Pasteura 1, 02-093 Warsaw, Poland

**Keywords:** bilirubin, peroxidation, peroxyl radicals, micelles, liposomes, antioxidant, radicals

## Abstract

Bilirubin (BR), a product of heme catabolism, plays a critical role in biological systems. Although increased levels of BR result in hyperbilirubinemia or jaundice, there is increasing evidence that lower concentrations substantially decrease the risk of oxidative stress-mediated diseases due to antioxidant functions of BR. We studied the radical-trapping ability of BR in two model systems, micellar and liposomal, at a broad pH range. At pH < 6.0, BR behaves as a retardant; however, at pH ≥ 6.0, BR becomes strong radical trapping antioxidant, with rate constants for reaction with lipidperoxyl radicals (*k*_inh_) within the range from 1.2 × 10^4^ M^−1^ s^−1^ to 3.5 × 10^4^ M^−1^ s^−1^, and in liposomal system, the activity of BR is comparable to α-tocopherol. This transition is likely facilitated by the ionization of carboxyl groups, leading to a conformational shift in BR and improved solubility/localization at the water/lipid interface. This is the first experimental evidence of the role of pH on the antioxidant activity of bilirubin, and the observed pH-dependent radical-trapping ability of BR holds practical significance, particularly in jaundice treatment where light therapy targets the skin’s weakly acidic surface. Minor adjustments toward neutral or alkaline pH can enhance radical-trapping action of BR, thereby mitigating oxidative stress induced with blue or violet light exposure.

## 1. Introduction

**Figure 1:**
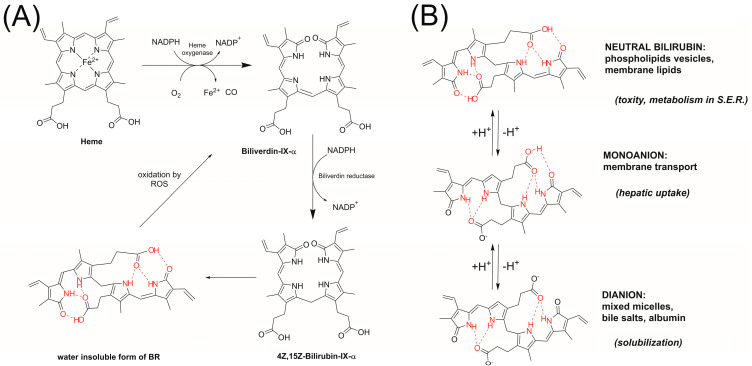
(**A**) Degradation of heme to biliverdin and formation of bilirubin. (**B**) Bilirubin is water insoluble, but deprotonation of neutral (diacid) BR into monoanion or dianion increases the interactions with membranes, micelles, and cytosolic binding proteins [2]. Hydrogen bonds are marked in red. Please notice that N–H–O hydrogen bonds exist even after complete deprotonation.

Bilirubin (BR) is an end-product of catabolism of heme (see Figure 1A), and major fraction of BR origins from degradation of hemoglobin in senescent erythrocytes, proceeding via two consecutive reactions, conversion of heme into biliverdin (catalyzed with heme oxygenase), which is reduced by biliverdin reductase to tetrapyrrole pigment.

Two dipyrolle fragments in BR are connected with methylene bridge, and the whole molecule in its neutral (non-ionized) BR has a folded ridge-tile shape conformation [1,2]. The carboxyl groups form strong hydrogen bonds (HB) with N–H and C=O moieties of terminal pyrrinone rings, and have overlapping p*K*_a_’s, 8.1 and 8.4 [3]. Thus, at physiological pH 7.4, a large fraction of BR is neutral. Even after deprotonation of the first carboxyl, strong internal hydrogen bonds make the BR structure highly hydrophobic; see Figure 1B. Solubility of BR in water is approximately 70 nM, and can be increased using alkalinization of the media or with the addition of strong H-bonding agents, being competitive to internal hydrogen bonds [4]. This mechanism of BR solubilization is effective in an organism, and 99% of BR in plasma is bound to serum albumin, and such indirect (or unconjugated, bound) BR is transported to the liver. In hepatocytes, upon interaction with ligandin, BR is transported to endoplasmic reticulum where it undergoes enzymatic conjugation with glucuronic acid to form direct (conjugated) BR. Unconjugated unbound BR is called free serum albumin, and its concentration is reported to be 8.3–13.1 nM [5]. The transport of conjugated BR into bile and its secretion into the blood is facilitated by ATP-dependent transporters (MRP2 and MRP3). Approximately 250–300 mg bilirubin/day is formed in a normal adult [5], and the total concentration of all kinds of BR in serum is within 5–15 μM.

Imbalance in BR production and elimination at any metabolic step has serious consequences. Increased red blood cell destruction or impaired conjugation is a cause of increased level of unconjugated BR, while hepatocellular damage or biliary tract obstructions produces an elevated level of conjugated BR. Both kinds of dysregulation of the metabolic/transport pathways are manifested as hyperbilirubinemia (serum concentration above 1 mg/dL) or jaundice (above 2–3 mg/dL) [6]. Unconjugated BR can cross the brain–blood barrier (BBB), and its accumulation might cause irreversible neurological damage, like acute bilirubin encephalopathy (ABE) and kernicterus spectrum disorders (KSDs). Toxic effects of BR deposited in the brain include inhibition of DNA synthesis, damage of mitochondrial metabolism, alteration of carbohydrate metabolism, and other undesirable effects [6].

**Figure 2:**
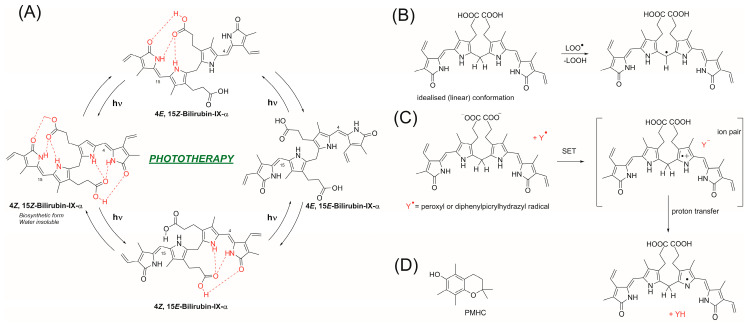
(**A**) Mechanism of reversible photo-induced changes of BR structure and its solubilization as proposed described in review by Nocentini et al. [6]. (**B**) Mechanism of hydrogen atom transfer (HAT) from central methylene to peroxyl radical (LOO^•^). (**C**) Reaction of anion form of BR with electron deficient radical Y^•^ (peroxyl of diphenylpicrylhydrazyl), as proposed in [9,10]. For clarity, a linear conformation of neutral or ionized bilirubin is presented in panels (**B**–**D**). Structural formula of 2,2,5,7,8-pentamethylchroman-6-ol (abbreviated as PMHC, an analogue of α-tocopherol).

Although the process of crossing the BBB is slow in adults, in the first days of living, the BBB is more permeable, and newborns are especially vulnerable to BR-dependent neurological damage. The molecular mechanism of cyto- and neuro-toxicity of BR underlying the oxidative stress is caused by superoxide, because BR is an inhibitor of cytochrome-c oxidase. Other mechanisms include the disruption of the mitochondrial membrane and activation of caspase-3-triggering apoptosis. In order to avoid harmful consequences, infants with neonatal hyperbilirubinemia are subjected phototherapy, as blue or white light (and, recently, green light is postulated [7]) converts BR into a more polar, less H-bonded structure (see Figure 2) [4], although some of them also have a toxic effect on human neurodevelopment [8]. High concentrations of BR are correlated with an increase in the oxidative stress in newborns, although such a correlation might result from other factors like decomposition of the hem and release of iron ions (see Figure 1A), and the pro-oxidation effect of phototherapy might be a side-effect of this common treatment applied to reduce hyperbilirubinemia [6].

High concentrations of BR are toxic, but small non-pathologic amounts are profitable for the organism, and may even have therapeutic effects on cardiovascular problems, stroke, and metabolic syndrome [6]. The organism economy, in a perfect way, turns the waste product of heme decomposition into a powerful antioxidant, anti-inflammatory agent, and immunomodulator. Due to this reason, mild hyperbilirubinemia is considered as an attractive approach to prevent many diseases mediated by oxidative stress and inflammation [11], and potential therapeutic applications have recently been reviewed by Adin [12].

Peroxidation of lipids is a main process resulting in deterioration of both artificial and natural self-organized lipid systems (emulsions, biomembranes, LDL particles, etc.), and since this process is mediated by peroxyl radicals and occurs within the non-polar phase, the reaction of bilirubin with peroxyl radicals has attracted much attention and has resulted in a number of publications. Stocker and coworkers suggested that BR reacts with peroxyl radical via Hydrogen Atom Transfer from the C–H bond at central C-10 position of tetrepyrrole molecule with the formation of a carbon-centered radical [13], the mechanism represented in Figure 2B. Bearing in mind that C-centered radicals react very fast with O_2_, making them ineffective antioxidants, the authors justified the antioxidant effect of BR by the reversibility of O_2_ addition, and such a hypothesis was supported by the observation that antioxidant activity of bilirubin increased when the experimental concentration of oxygen was decreased from 20% to 2%. The same authors observed a much higher antioxidant activity of BR in the liposomal system in comparison to homogeneous solution, and such an effect was rationalized as it was due to increased local concentration of hydrophobic BR in the same phase as lipidperoxyl radicals. Barclay and coworkers [9] also reported very weak antioxidant activity of BR in nopolar medium, which turned into strong inhibiting properties (similar to α-tocopherol and its analogues) in lipid bilayers and in SDS micelles at pH 7.4. The authors proposed that such different behavior in polar and dispersed systems is due to electron transfer mechanism manifested in polar medium; see Figure 2C. They also compared the activity of dimethyl esters of BR and biliverdin with the activity of several synthetic dipyrrinones bearing free or methylated N–H functionality [10]. Those studies, carried out in nonpolar solvent, together with theoretical calculations, suggested Hydrogen Atom Transfer (HAT) from N–H to peroxyl radicals. Alternatively, BR might undergo electron transfer followed by fast deprotonation (see Figure 2C). Both mechanisms result in the formation of pyrrolyl radical with a very weak C–H bond in central methylene group (40.9 kcal/mol) and after HAT to another peroxyl radical, double bond is established. Such conversion of BR into biliverdin consumes two radicals, however, further antioxidant action is possible because biliverdin can trap next peroxyl radicals. Some BR derivatives and smaller molecules structurally similar to part of BR were also studied in biphasic systems with the conclusion that the main reason of different antioxidant behavior of BR in polar and non-polar solvents is due to unfolding the “ridge-tile” conformation of free BR, and some of its six hydrogen bonds are changed from internal into external HB upon polar H-bond acceptors, affecting localization of BR and its ability to trap peroxyl radicals [14]. For similar reasons, the results of studies of BR derivatives must be considered with caution; for example, two carboxyl groups are crucial for the formation of conjugated bilirubin during the liver’s processing, and a small change like esterification of two carboxyls make a big difference between native BR and its model analogs. Although the kinetic studies in artificial systems might explain the increased stoichiometry, in the cells, a phenomena of efficient trapping can be explained by the enzymatic recovery of intracellular BR with biliverdin reductase; see Figure 1A [15,16]. In this way, BR is a very effective antioxidant with a very small value of parameter EC_50_ = 11.4 ± 0.2 nM reported in endothelial cells exposed to peroxyl radicals, and such EC_50_ is comparable to concentration of free serum BR [5].

Polarity, charge of bilayer, and pH are very important for the localization of phenolic antioxidants and, consequently, their ability to trap peroxyl radicals [17,18,19]. Taking into account the crucial role of BR structure on the solubility and localization, we can put out the hypothesis that protonation/deprotonation equilibria is important for the antioxidant activity of BR during peroxidation of dispersed lipid in water systems, and we decided to check the pH dependence of the ability of BR to trap peroxyl radicals. This overlooked factor can be important, as a slight change in pH could convert some molecules of BR into antioxidant, minimizing the oxidative stress, as well as protecting against the undesired side-effects of phototherapy.

## 2. Materials and Methods

Chemicals. Initiator 2,2′-Azobis(2-amidinepropane) dihydrochloride (ABAP; 97%, Sigma–Aldrich, Saint Louis, MO, USA), bilirubin (BR; 98%, Sigma–Aldrich, Merck group, Germany), Triton X-100 (polyethylene glycol p-(1,1,3,3-tetramethylbutyl)-phenyl ether, 98%, Carl Roth, Karlsruhe, Germany), methyl linoleate (MeLin; 99%, Sigma–Aldrich), and 2,2,5,7,8-pentamethylchroman-6-ol (PMHC; 99%, Sigma–Aldrich), 1,2-dimyristoyl-sn-glycero-3-phosphocholine (DMPC; 99%, Avanti Polar Lipids Inc., Birmingham, AL, USA) were used as received. Buffers were prepared from inorganic components (acetate buffer for pH 4 and 5, phosphate buffer tor pH 6–8, and borate buffer for pH 9), and the ionic strength was kept with KCl.

Preparation of Micelles. This was described in our previous kinetic studies [17,20,21]. In total, 5.5 mL of an aqueous solution of Triton X-100 (16 mM) and 10 µL of methyl linoleate were placed in a glass test tube and, after one minute of mixing on a Vortex, 5.5 mL of an aqueous buffer solution with a known pH was added and mixed again on a Vortex for one minute. The final concentration of MeLin and Triton X-100 in the micellar system was 2.74 mM for lipid and 8 mM for surfactant.

Preparation of Liposomes (Large Unilamellar Vesicles, LUVs). LUVs were obtained from multilamellar vesicles (MLVs) by a previously described extrusion procedure [21]. Preparation of liposomes began with dissolving 65.3 mg of lipid (DMPC) in a small amount of chloroform (approximately 1.5 mL). After mixing the solution for one minute on a Vortex, 4 µL of methyl linoleate was added, and the mixture was again mixed for one minute on a Vortex. Then, chloroform was slowly evaporated on a rotary evaporator to form a thin lipid film on the inner walls of the flask. Flasks containing the lipid films were dried overnight at room temperature under vacuum. The dried liposomes were suspended in 4.4 mL of an aqueous buffer (the same inorganic components as those for micellar systems). The final concentration of lipids was 2.74 mM MeLin and 20.2 mM DMPC. The liposome suspension was extruded (21 extrusions) by passing the mixture through a membrane with a pore diameter of 0.1 µm in an Avanti mini extruder (Avanti Polar Lipids Inc.). Based on the DLS method, the size distribution of LUVs was determined, and it was 170 ± 45 nm (in agreement with previously prepared LUVs [21]).

Methodology of Autoxidation Measurements. The uptake of dissolved oxygen during lipid peroxidation was carried out at 37 °C using a Yellow Spring Instrument 5300A Biological Oxygen Monitor (YSI, Yellow Springs, OH, USA) connected with two Clark-type oxygen electrodes in a temperature-controlled water bath. In order to protect BR against light induced change of conformation (see Figure 2) during the kinetic measurements, the system was covered with aluminum foil. The sample (5 mL of emulsion or 2 mL for liposomal system) was placed in a small chamber equipped with a magnetic stirrer, and the electrode was mounted inside the chamber, and peroxidation was initiated with the injection of an aqueous 0.5 M solution of ABAP. After oxygen content in the sample decreased to 90–80% of starting value, 10 µL of an ethanolic solution of the antioxidant at a concentration of 0.5 mM was added using a glass micro-syringe. In such prepared samples, the concentrations of individual substances were as follows: 2.74 mM methyl linoleate, 8 mM Triton X-100, 10 mM ABAP, and 1.0 × 10^−6^ M antioxidant (BR or PMHC). In the experiments, PMHC was used as a standard phenolic antioxidant. PMHC and α-tocopherol exhibited antioxidant activity due to 6-hydroxychroman; the difference is that PMHC lacks a phytyl chain (that is inert in radical scavenging). Due to its simple structure, PMHC is a perfect model molecule mimicking tocopherols, flavanes, isoflavanes, and some pharmaceutics containing chromanol as a structural feature.

BR is sparingly soluble in solvents mixable with water, but it is readily soluble in alkaline solutions. The addition of 100 µL of 0.1 mM NaOH to 5 mL of BR suspended in ethanol makes the solution clear and orange. We checked that, after the addition of 10 µL of such alkaline solution to 5 mL of phosphate buffer pH = 7.0, its pH value changed slightly (by 0.05 units). Therefore, for further studies, 5 mL of ethanol stock solution of bilirubin (0.1 mM) were alkalized with 100 µL of 0.1 M NaOH. This procedure is similar to the one used by Barclay [9].

## 3. Results and Discussion

Experiments carried out in model systems (homogeneous solutions or dispersed lipid/water systems) subjected to peroxidation provide an essential quantitative information on the ability a studied compound to break a chain process mediated with lipidperoxyl or alkylperoxyl radicals. Therefore, among many studies of BR, the ones focused on the kinetics of lipid peroxidation provide the most comprehensive description of BR as chain-breaking (radical trapping) antioxidant. Briefly, the process is initiated by free radicals In^•^ generated during thermal decomposition of initiator (In)_2_, usually a diazocompound. Primary formed In^•^ radicals after fast reaction with molecular oxygen are converted into peroxyl radicals, InOO^•^ (reaction (1)), which are able to abstract hydrogen atom from lipid molecule LH (reaction (2), initiation, with rate *R*_i_). Newly formed alkyl radical L^•^ triggers the propagation cycle of consecutively occurring very fast addition of oxygen (reaction (3), with *k*_O2_ > 10^8^ M^−1^ s^−1^) and much slower abstraction of hydrogen from LH (reaction (4)). Reaction (4) is the rate-limiting step, with rate constant *k*_p_ depending on the number of accessible H atoms at *bis*-allyl positions and on the physical nature of the system being peroxidized (solution, bilayers, emulsions, etc.). For lipids containing linoleate derivatives, like linoleic acid, methyl linoleate, and phospholipids containing linoleic acyl group, *k*_p_ is from 15 to 100 M^−1^ s^−1^, see data collected in Supplementary Material to ref. [17].

(In)_2_ → 2*f* In^•^ → 2*f* InOO^•^ (1)

InOO^•^ + L-H → InOOH + L^•^       *R*_i_ (2)

L^•^ + O_2_ → LOO^•^       ; *k*_O2_ (3)

LOO^•^ + L-H → LOOH + L^•^       ; *k*_p_ (4)

Common antioxidants (AoxH) inhibit peroxidation of organic materials by trapping peroxyl radicals (reactions (5) and (6)):

LOO^•^ + AoxH → LOOH + Aox^•^        *k*_inh_ (5)

LOO^•^ + Aox^•^ → non radical products (6)

For effective antioxidants, the rates of reactions (5) and (6) are much faster than the rate of propagation (4), and the overall rate of autoxidation is suppressed until all antioxidant molecules are consumed. For monophenolic antioxidants (AoxH = PhOH) the stoichiometric factor *n* (the number of radicals trapped by one molecule of antioxidant) is generally 2, which is a net effect of reactions (5) and (6), but usually the efficiency of trapping is smaller, and *n* < 2.

Time τ from the injection of antioxidant to the end of the suppressed process is called the induction period, and the rate of inhibited process is expressed by:

(7) $$R_{inh}=-\frac{d\left[O_{2}\right]}{dt}=\frac{k_{p}\left[\mathrm{LH}\right]R_{i}}{nk_{\mathrm{inh}}{\left[\mathrm{AoxH}\right]}_{t}}$$

where [Aox-H]_t_ is the diminishing concentration of the antioxidant. Rate of initiation, *R*_i_, can be easily determined from the length of the induction period obtained for the known starting concentration of antioxidant [AoxH]_0_:

(8) $$R_{i}=n\;{\left[\mathrm{AoxH}\right]}_{0}/\tau$$

A convenient model AoxH for such experiments is α-tocopherol or its analogue PMHC (2,2,5,7,8-pentamethyl-6-hydroxychroman; see Figure 2D), for which *n* = 2.0.

When parameters [LH], *R*_i_ and [Aox-H]_τ_ are known, precise measurements of oxygen uptake can give *k*_p_/*nk*_inh_ ratio, and the calculation of *k*_inh_ would be easy from Equation (7). However, *n* is not always known, and [AoxH]_τ_ differs from initial concentration [AoxH]_0_, and thus, the integral form of 7 is used [22,23]:

(9) $$-\Delta {\left[O_{2}\right]}_{t}=k_{p}\left[\mathrm{LH}\right]ln\left(1-t/\tau \right)/k_{\mathrm{inh}}$$

and for t < τ a plot of ∆[O_2_]_t_ vs. ln(1 − t/τ) gives a straight line of slope *k*_p_[LH]/*k*_inh_ from which *k*_inh_ is obtained using a substrate with a known *k*_p_ (herein 36 M^−1^ s^−1^ for oxidation of methyl linoleate in emulsion [24], and 41 M^−1^ s^−1^ in liposomes [23]).

### 3.1. Results Obtained in Micellar Systems

**Figure 3:**
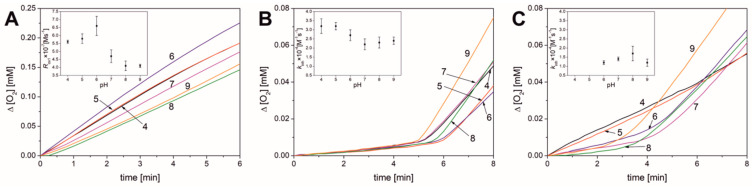
Oxygen consumption curves for autoxidation of methyl linoleate emulsion initiated with 10 mM ABAP over the entire pH range (4.0−9.0) at a temperature of 37.0 °C (**A**) without antioxidant (inset: plot of rate of oxidation versus pH); (**B**) with 1.0 µM PMHC (inset: plot of *k*_inh_ versus pH); (**C**) with 1.0 µM BR (inset: plot of *k*_inh_ versus pH).

Micelles were formed using Triton X-100 doped with methyl linoleate (final concentrations were 8 mM Triton X-100 and 2.74 mM methyl linoleate). The oxygen consumption curves presented in Figure 3A for uninhibited oxidation are linear, and their slopes are relatively similar, regardless of the pH applied. Therefore, it can be concluded that the values of uninhibited peroxidation rates fall within the range of (4.1–6.6) × 10^7^ M s^−1^ over the entire pH range; see Table 1 and inset to Figure 3A. This observation is important for further research, as it signifies that the kinetics of oxidation in the model lipid micellar system is not sensitive to pH changes.

The oxygen consumption curves for emulsions containing PMHC (Figure 3B) show a characteristic pattern for processes effectively inhibited with a good antioxidant, with a distinct induction period, where the rate of oxidation is significantly lower. After the complete PMHC is exhausted, the peroxidation rate begins to increase. Table 1 presents the kinetic parameters determined from the curves, such as induction period length (τ), inhibited process rate (*R*_inh_), and post-inhibition oxidation rate (*R*_ox2_). The induction period is clearly visible, and does not differ significantly at different pH values, ranging from 5.4 to 6.5 min. Parameters describing the course of peroxidation in the presence of PMHC show slight differences within the investigated pH range. The experiment with PMHC is crucial for further research because, based on the value of τ and concentration of the used antioxidant (1.0 µM), *R*_i_ can be determined (see Equation (8)). These values are also presented in Table 1, and regardless of the pH, *R*_i_ = 5.6 ± 0.6 nMs^−1^.

Knowing the values of *R*_i_, *R*_inh_, and *R*_ox_, the length of the kinetic chain of peroxidation (ν) can be calculated. This parameter expresses the number of propagation cycles initiated by one radical before the chain terminates. From the ν_ox_ values presented in Table 1, it can be inferred that for the uninhibited process, one initiating radical causes the oxidation of 39 (ν_ox1_ at pH 4) to 73 (ν_ox1_ at pH 9) lipid molecules. However, in the presence of an inhibitor, the propagation chain is approximately ten-fold smaller (3.7 < ν_inh_ < 5.4). Another significant conclusion is that even in the presence of 1 µM PMHC, the reaction still follows a chain mechanism (ν_inh_ > 3), and, therefore, the methodology for determining kinetic parameters can be applied in accordance with the assumptions of steady-state theory.

Based on Equation (9), the rate constants for the reaction of PMHC with peroxyl radicals (reaction (5)) were calculated. The *k*_inh_ values compiled in Table 1 are comparable to the literature values, ranging from 1.9 × 10^4^ M^−1^ s^−1^ to 2.4 × 10^4^ M^−1^ s^−1^ for the pH 4.0–9.0 [19], and are close to two values (2.0 × 10^4^ M^−1^ s^−1^ at pH 4.0 and 2.2 × 10^4^ M^−1^ s^−1^ at pH 7.0) reported by Grębowski et al. [17].

Compared to PMHC, the oxygen consumption curves for the BR-inhibited process at a concentration of 1 µM (Figure 3C) show a different shape. Firstly, at pH 4.0 and 5.0, the oxidation is slower than non-inhibited process, but the curves are linear, without induction period, and such linear plots are typical for retardants [19]. The lengths of the kinetic chain during retardation at pH 4 and 5 are 22 and 35, respectively. Since BR did not exhibit an induction period at pH 4.0 and 5.0, it was not possible to determine τ, *n*, and *k*_inh_ for these pH values. However, when pH is increased above 5, the induction period becomes clearly visible. After all BR is consumed during induction period, the peroxidation rate starts to increase. Parameters determined for peroxidation inhibited with BR show slight differences within the investigated pH range (Table 1). The stoichiometric coefficient *n* at pH 6–9 falls within the range of 1.7 to 2.0. These values indicate that bilirubin is capable of removing nearly the same amount of peroxyl radicals as PMHC.

### 3.2. Results Obtained for Liposomal Systems

**Figure 4:**
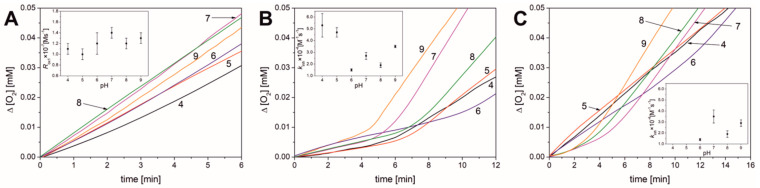
Oxygen consumption curves for autoxidation of methyl linoleate in liposomal DMPC system initiated with 10 mM ABAP, over the entire pH range (4.0−9.0) at a temperature of 37.0 °C (**A**) without antioxidant (inset: plot of rate of oxidation versus pH); (**B**) with 1 µM PMHC (inset: plot of *k*_inh_ versus pH); (**C**) with 1 µM BR (inset: plot of *k*_inh_ versus pH).

Liposomes were prepared using 1,2-dimyristoyl-sn-glycero-3-phosphocholine (DMPC) doped with methyl linoleate (final concentrations were 22 mM DMPC and 2.74 mM linoleate). A detailed description of liposome preparation is presented in Section 2. Figure 4A presents the kinetic traces for oxygen consumption during oxidation (initiated with 10 mM ABAP) of methyl linoleate in liposomal system over the pH range 4.0–9.0.

The rate of uninhibited peroxidation ranges from 1.0 × 10^−7^ to 1.4 × 10^−7^ M s^−1^ (see Table 2) and is four times lower than the value of *R*_ox_ in micellar systems, indicating that, under the same conditions and similar rate of initiation (*R*_i_), the peroxidation is propagated slower in liposomal system than in the micellar systems, in agreement with our previous publication [17].

When PMHC is added to the liposomal system, a distinct induction period can be observed, ranging from 4.8 to 8.2 min (see Figure 4B), and after the antioxidant is completely consumed, peroxidation accelerates. Parameters τ, *R*_inh_, and *R*_ox2_ are listed in Table 2. Based on the value of τ and the concentration of the used antioxidant (1 µM each time), the rate initiation, *R*_i_, can be determined from Equation (8). The τ values obtained for PMHC were used for calculation of initiation rate, giving R_i_ = 5.4 ± 1.1 nMs^−1^ within the whole pH range. Thus, in the liposomal system, the initiation process also does not depend on pH. In the presence of 1 µM PMHC, the reaction still follows a chain mechanism (ν_inh_ > 3), and the *k*_inh_ values calculated for this antioxidant in liposomal system are comparable to the literature values [17], with the lowest *k*_inh_ at pH 6–8 and two- or three-fold higher in the lowest and highest pH.

Antioxidant behavior of BR in liposomes is more pH-dependent than in the micellar system. However, even here, at pH 4.0 and 5.0, the curves form straight lines, showing no induction period. At pH 6, the curve has an intermediate course: there is a deviation from a straight line and a very gentle bend between the eighth and eleventh minutes after bilirubin injection. The kinetic profile begins to resemble a curve typical for a sample containing an antioxidant, but the slowdown of the process is very slight (ν_inh_ = 12 vs. ν_ox_ = 18) and the induction period is not clearly visible. The kinetic profile noticeably changes with increasing pH, and, in the range from 7.0 to 9.0, inhibition can be easily observed; the induction periods were determined and are listed in Table 2. The above observations suggest that for liposomal systems, BR exhibits antioxidant properties at pH 6.0–9.0. Parameters *n* indicate that one BR molecule is able to reduce from 1.3 to 2.3 peroxyl radicals.

### 3.3. Influence of pH on the Mechanism of Bilirubin Action

Kinetic parameters for the reaction of BR with lipidperoxyl radicals in dispersed lipid/water systems at pH 7.0 determined here are 1.4 × 10^4^ and 3.5 × 10^4^ M^−1^ s^−1^ in DMPC, and both values are comparable to *k*_inh_ = 1.1 × 10^4^ M^−1^ s^−1^ obtained for BR conjugates with sodium taurocholate in SDS micelles and in DLPC liposomes at pH 7.4 [14]. Very similar values of *k*_inh_ (from 4.7 to 5.2 × 10^4^ M^−1^ s^−1^) were determined for BR without taurocholate in SDS at pH 7.4 [9]. Stoichiometric coefficients obtained in our systems at pH 7.0 were also similar to the literature ones *n* = 1.8 [14] and *n* = 1.5 [9]).

The most intriguing observation is that, in both micellar and liposomal systems, in a relatively acidic environment (pH 4 and 5), BR does not act as an antioxidant, but the behavior is typical for retardants. However, BR turns into an antioxidant at pH ≥ 6, with clear induction periods for which the rate constants can be calculated and, indeed, in the emulsion system, *k*_inh_ for BR is half of the value of *k*_inh_ obtained for analogue of α-tocopherol. In the liposomal system, BR becomes even more active, as, at pH ≥ 6, the values of *k*_inh_ for BR and for PMHC are close to each other. This is the first experimental evidence of the role of pH on the antioxidant activity of bilirubin.

The initial hypothesis adopted to explain the lack of antioxidant activity of BR at pH < 6 was the possibility of protonation of bilirubin fragments. This would result in a change in electron density in the molecule and an increase in ionization potential (IP) or an increase in the bond dissociation energy of N–H (BDE_N–H_). The former parameter is crucial for the SET mechanism proposed in the literature, while BDE is important for the HAT mechanism. However, direct protonation of pyrrolic fragments is not possible because the pyrrole ring is a very weak base and its conjugated acid has a negative p*K*_a_ = −3.8 [25,26], much lower than for protonated aniline, pyridine, or piperidine (for which p*K*_a_’s are 4.6, 5.2, and 11, respectively [27]). Protonation of pyrrole would cause a loss of aromaticity because, during protonation, the electrons of the lone pair of nitrogen must form a new bond, no longer interacting with the π electrons of the aromatic ring. None of the four rings (two pyrroles and two pyrrolinones) of BR should exhibit stronger basicity than the model unsubstituted pyrrole. Therefore, any acid-base equilibria for BR can only be attributed to the carboxyl groups. Short-chain aliphatic carboxylic acids have p*K*_a_ values lower than 5. The early measurements for BR indicating p*K*_a_ from 4.3 to 5.9 were carried out in strong hydrogen-bonding solvents, such as DMSO. During these measurements, BR no longer had its native structure [28]. Some values below 6 were also reported for BR in water with a small quantity of DMSO (^13^C NMR titration) [29,30]. Mukerjee and Ostrow, in a series of reviews, criticized the low values of p*K*_a_ [2,3,31]. They proposed to estimate acidity on the basis of results of measurements of partition coefficients, studies of BR conjugates with biomolecules, association and aggregation, and spectroscopic studies of BR conformation at different pH levels, and proposed that BR in the form of self-aggregates exhibits a p*K*_a_ of 6.4, while two p*K*_a_ values of 8.12 and 8.44 are assigned to the non-associated form. The authors explain such low acidity as an effect of steric crowding in the microenvironment of carboxyl groups and carboxylate anions, caused by the presence of hydrogen bonds formed by COOH groups, strong solvation of carboxylate anions, and the resulting stiffening of the molecule (inhibited rotation) [2]. Therefore, in physiological pH, the dominant form of BR would be the neutral molecule or monoanion, and such a form should be more easily transported into hepatocytes.

The considerations on the acidity of carboxyl groups in BR can be used as arguments converging with the observed relatively strong antioxidant properties in both heterogeneous systems at pH > 6.0, with the highest values of *k*_inh_ at pH 7 and 8. Since pyrrole and pyrrinone rings do not change their protonation status at pH range 4–9, the enhancement of antioxidant activity is not an effect of direct acid/base catalysis in the reaction site, but must be connected with the ionization status of the carboxyl group. One of the possible explanations is that, after deprotonation of COOH, the lactam C=O group from terminal pyrrinone rings becomes available for water molecule, and the ridge-tile structure of BR is distorted [2]. Similar enhancement of antioxidant activity of BR was observed when carboxyl groups were esterified [10] because esterification makes molecules more open and more active towards peroxyl radicals. The hypothesis of conformation dependent antioxidant activity of BR was already applied to explain the antiradical activity of BR in polar solvents and in heterogeneous liposomal and micellar systems [9,13,32]. A similar explanation can be employed for the pH-dependent activity of BR observed in our experiments, and such a rationale is supported by the observation that, for increasing pH, the hydrophobic aggregates of BR turn into monomeric BR [33]. Thus, peroxyl radicals have better access into such an open molecule of BR. As can be seen in Figure 1B, in monoanion, each N–H moiety is still internally H-bonded; however, in contrast to the intermolecular hydrogen bond, which excludes the H atom abstraction [34,35], the internal hydrogen bonding does not block the N–H or O–H bond and does not exclude HAT, although the abstraction is slower [36,37]. As pyrroles are an energy-rich species, an easier and sterically less demanding mechanism would be a sequence of electron transfer followed by proton transfer. The process is presented in Figure 2C with the ion pair as intermediate, and strongly acidic radical cation immediately loses the proton, which is transferred directly or indirectly (via solvent molecule) to the Y^−^ counter ion.

The third mechanism, with central –CH_2_– in BR as an active H atom donor [13], was verified by Barclay et al. [10]. They calculated the C–H bond strength for dipyrrinone derivative to be ca. 70.1 kcal/mol compared to 84.7 kcal/mol for N–H bond. However, the authors pointed out that the ridge-tile conformation of BR hinders HAT from central methylene, and the real bond strengths would be much higher because an additional 15–20 kcal/mol is needed for stabilization of the formed –^•^CH– radical of BR. Therefore, such energy cost makes this mechanism less competitive to other two mechanisms.

Regardless of the mechanistic consideration, it must be pointed out that with increasing pH, a new negative charge appears which affects the conformational state of BR (resulting in monomeric or aggregated form) and, as a consequence, the interaction and localization at the water/lipid boundary [2]. The accessibility of antioxidant for peroxyl radicals operating in the lipid phase is of crucial importance for antioxidants [18]. Thus, the pH-dependent ability of BR to form complexes with plasma proteins and bile components (salts, micelles, phospholipids) is well in line with the observation that an increase from pH 5 to pH 6 and above makes a big difference in the radical trapping ability of BR. On the other side, the effect of the pH-dependent activation of BR is more subtle than the simple concentration-dependent antioxidant/prooxidant activity of BR in cellular systems. For example, Bianco and coworkers [38] demonstrated in four cell lines that the intracellular unconjugated BR at concentration around 7 ng/mg protein had antioxidant activities while, at a concentration above 25 ng/mg protein, the antioxidant effect changes into pro-oxidant and cytotoxic effects. Herein, a change of pH from 5 to 7 must be correlated with the changing mechanism and accessibility of antioxidant to peroxyl radicals, but not to a very high increase of concentration which would allow the conversion of BR into prooxidant.

The observed pH-dependent antioxidant activity of BR in the micellar and liposomal system cannot be simply translated to cellular systems. However, pH and local pH can vary between the cells, tissues, and organs. The most spectacular example of extreme pH is the stomach, but pH in other organs and tissues can vary with three units difference, which might be of crucial importance for the effectiveness (and mechanism) of radical trapping. The effect of BR activation when passing from pH 5 to pH 6 and higher might be of practical importance, because one method of treating jaundice involves strong exposure to blue or violet light, which is a source of reactive oxygen species and triggers a massive oxidative stress. During such therapy, the light operates on the skin surface, and the skin pH is weakly acidic, and a slight change in pH from 5.5 to values of 6–7.4 (which can be achieved with creams, including cosmetics based on emulsions and liposomes) can lead to an increase in the protective, radical trapping action of BR. In this way, part of the bilirubin would undergo photodecomposition, but, at higher pH, an additional fraction of BR would act against induced oxidative stress and the associated further peroxidation of biomolecules.

## 4. Conclusions

The results presented here provide the first comprehensive comparison of the radical trapping ability of bilirubin in two model systems (micellar and liposomal) over a relatively wide pH range. At pH < 6.0, bilirubin acts as a retardant, and its low activity may be the result of its closed ridge-tile structure (involving non-dissociated carboxyl groups). At pH > 6.0, BR turns into stronger antioxidant, comparable to the analogue of α-tocopherol. We suppose the observed enhancement of radical trapping ability is due to ionization of carboxyl group(s) and disruption of hydrogen bonds resulting in conformational shift in bilirubin, increased solubility/localization at the lipid–water interface and improvement of accessibility of bilirubin molecule to lipidperoxyl radicals.

The observation of pH-dependent ability of bilirubin to trap radicals in dispersed lipid–water systems can be of practical importance. Bilirubin activation occurs between pH 5 and 6. However, the light therapy during jaundice treatment targets the skin’s weakly acidic surface (pH 5.5), thus, minor pH adjustments to 6–7.4 (achievable with pH-modifying creams, including those containing liposomes) can enhance BR’s radical-trapping action, resulting in minimizing oxidative stress induced with blue or violet light.

## Figures and Tables

**Table 1:** Parameters determined for initiated autoxidation of methyl linoleate emulsion containing 1.0 µM PMHC and 1.0 µM BR at pH 4.0–9.0: induction period length (τ), autoxidation initiation rate (R_i_), inhibited autoxidation rate (R_inh_), inhibition rate constants (*k*_inh_), rates of non-inhibited and post-inhibition process (*R*_ox1_ and *R*_ox2_, respectively), and the kinetic chain length *^a^*^,*b*^ of peroxidation (ν_ox1_, ν_inh_; ν_ox2_).

Parameter	pH					
	4.0	5.0	6.0	7.0	8.0	9.0
			none			
*R*_ox1_ × 10^7^ [M s^−1^]	5.6 ± 0.1	5.8 ± 0.3	6.6 ± 0.6	4.7 ± 0.4	4.1 ± 0.3	4.1 ± 0.1
*ν*_ox1_ *^b^*	90 ± 2	110 ± 6	122 ± 11	86 ± 7	80 ± 6	69 ± 2
			PMHC			
*τ* [min]	5.4 ± 0.1	6.3 ± 0.3	6.1 ± 0.4	6.1 ± 0.1	6.5 ± 0.4	5.6 ± 0.3
*R*_i_ × 10^9^ [M s^−1^]	6.2 ± 0.2	5.3 ± 0.2	5.5 ± 0.4	5.5 ± 0.1	5.1 ± 0.3	6.0 ± 0.3
*R*_inh_ × 10^8^ [M s^−1^]	2.3 ± 0.2	2.5 ± 0.1	3.0 ± 0.7	2.7 ± 0.5	2.7 ± 0.4	3.3 ± 0.5
*R*_ox2_ × 10^7^ [M s^−1^]	2.5 ± 0.1	2.3 ± 0.2	2.2 ± 0.1	3.0 ± 0.1	3.3 ± 0.2	4.4 ± 0.4
*ν*_inh_ *^a^*	3.7 ± 0.3	4.7 ± 0.4	5.4 ± 0.8	5.0 ± 0.9	5.2 ± 0.9	5.4 ± 0.7
*ν*_ox_ *^b^*	39 ± 1	44 ± 3	41 ± 4	55 ± 2	65 ± 2	73 ± 4
*k*_inh_ × 10^−4^ [M^−1^ s^−1^]	3.2 ± 0.4	3.2 ± 0.2	2.7 ± 0.3	2.2 ± 0.3	2.3 ± 0.3	2.4 ± 0.2
			Bilirubin			
*τ* [min]	-	-	4.9 ± 0.2	5.4 ± 0.1	5.0 ± 0.3	4.5 ± 0.6
*n*	-	-	1.8 ± 0.1	2.0 ± 0.1	1.9 ± 0.1	1.7 ± 0.2
*R_inh_* × 10^8^ [M s^−1^]	11 ± 1	11 ± 1	5.8 ± 0.4	4.1 ± 0.8	3.5 ± 0.7	5.3 ± 0.4
*R_ox2_* × 10^7^ [M s^−1^]	1.4 ± 0.1	1.9 ± 0.1	2.5 ± 0.1	2.7 ± 0.3	2.4 ± 0.2	3.7 ± 0.3
*ν_inh_ ^a^*	22 ± 1 *^c^*	35 ± 3 *^c^*	10.7 ± 0.7	7.4 ± 1.4	6.7 ±1.4	8.8 ± 0.7
*ν_ox2_ ^b^*			46 ± 1	49 ± 6	46 ± 3	62 ± 4
*k_inh_* × 10^−4^ [M^−1^ s^−1^]	-	-	1.2 ± 0.1	1.4 ± 0.1	1.7 ± 0.4	1.2 ± 0.2

*^a^* ν_inh_ = R_inh_/R_i_. *^b^* ν_ox1_ = *R*_ox1_/*R*_i_ and ν_ox2_ = *R*_ox2_/*R*_i_. *^c^* ν_inh_ calculated for retarded process.

**Table 2:** Parameters determined for initiated autoxidation of liposomes containing methyl linoleate in the presence of 1.0 µM PMHC or 1.0 µM BR at pH 4.0–9.0: induction period length (τ), autoxidation initiation rate (R_i_), inhibited autoxidation rate (R_inh_), inhibition rate constants (*k*_inh_), rates of non-inhibited and post-inhibition process (*R*_ox1_ and *R*_ox2_, respectively), and the kinetic chain length *^a^*^,*b*^ of peroxidation (ν_ox1_, ν_inh_; ν_ox2_).

Parameter	pH					
	4.0	5.0	6.0	7.0	8.0	9.0
			None			
*R*_ox1_ × 10^8^ [M s^−1^]	11 ± 5	10 ± 1	12 ± 2	14 ± 1	12 ± 1	13 ± 1
*ν*_ox1_ *^b^*	17 ± 1	18 ± 1	30 ± 4	26 ± 1	27 ± 2	19 ± 1
			PMHC			
*τ* [min]	5.4 ± 0.1	6.1 ± 0.4	8.2 ± 0.6	6.2 ± 0.9	7.7 ± 0.2	4.8 ± 0.5
*R*_i_ × 10^9^ [M s^−1^]	6.1 ± 0.1	5.5 ± 0.4	4.1 ± 0.3	5.5 ± 0.7	4.3 ± 0.1	6.9 ± 0.7
*R*_inh_ × 10^8^ [M s^−1^]	1.8 ± 0.4	2.1 ± 0.5	2.5 ± 0.4	2.9 ± 0.2	2.7 ± 0.1	3.2 ± 0.5
*R*_ox2_ × 10^8^ [M s^−1^]	5.9 ± 0.4	6.3 ± 0.3	6.2 ± 0.5	14.8 ± 0.7	12.0 ± 0.9	13.6 ± 0.9
*ν*_inh_ *^a^*	3.0 ± 0.7	3.8 ± 0.7	6.2 ± 0.4	5.4 ± 1.0	6.2 ± 0.1	4.6 ± 0.3
*ν*_ox2_ *^b^*	10 ± 1	12 ± 1	15 ± 2	28 ± 4	28 ± 1	20 ± 2
*k*_inh_ × 10^−4^ [M^−1^ s^−1^]	5.3 ± 1.0	4.7 ± 0.4	1.5 ± 0.1	2.7 ± 0.3	1.9 ± 0.2	3.5 ± 0.1
			Bilirubin			
*τ* [min]	-	-	7.0 ± 0.7	5.1 ± 0.2	5.6 ± 0.4	4.0 ± 0.5
*n*	-	-	2.3 ± 0.2	1.7 ± 0.1	1.9 ± 0.1	1.3 ± 0.2
*R_inh_* × 10^8^ [M s^−1^]	5.5 ± 0.2 *^c^*	5.8 ± 0.2 *^c^*	4.9 ± 0.1	3.4 ± 0.6	4.4 ± 0.4	4.1 ± 0.5
*R_ox2_* × 10^8^ [M s^−1^]	5.6 ± 0.1	5.8 ± 0.4	7.5 ± 0.6	10.3 ± 1.4	10.9 ± 0.4	12.3 ± 0.6
*ν_inh_ ^a^*	9 ± 1 *^c^*	11 ± 1 *^c^*	12.1 ± 0.2	6.3 ± 1.1	10.2 ± 0.9	6.0 ± 0.7
*ν_ox2_ ^b^*	-	-	18 ± 2	19 ± 2	25 ± 1	18 ± 1
*k_inh_* × 10^−4^ [M^−1^ s^−1^]	-	-	1.4 ± 0.1	3.5 ± 0.6	1.9 ± 0.3	2.9 ± 0.3

*^a^* ν_inh_ = R_inh_/R_i_. *^b^* ν_ox1_ = *R*_ox1_/*R*_i_ and ν_ox2_ = *R*_ox2_/*R*_i_. *^c^* ν_inh_ calculated for retarded process.

## Data Availability

The raw data supporting the conclusions of this article will be made available by the authors on request.
